# Self-management of cognitive impairments: A qualitative study of cancer survivors’ coping strategies

**DOI:** 10.1007/s00520-025-09831-w

**Published:** 2025-08-08

**Authors:** Sunniva Brurok Myklebost, Karine Gjerde Bevan, May Aasebø Hauken, Ragnhild Johanne Tveit Sekse, Marit Therese Schmid, Stine Hope Spjeld, Glenn Fredrik Fletcher, Tine Nordgreen

**Affiliations:** 1 Division of Psychiatry, Haukeland University Hospital, Haukelandsbakken 2, 5009 Bergen, Norway; 2 Department of Global Public Health and Primary Care, Faculty of Medicine, University of Bergen, Bergen, Norway; 3 Center for Crisis Psychology, Faculty of Psychology, University of Bergen, Bergen, Norway; 4 Faculty of Health Sciences, VID Specialized University, Bergen, Norway; 5 Department of Welfare and Participation, Western Norway University of Applied Sciences, Bergen, Norway

**Keywords:** Cognitive impairment, Cancer survivors, Self-management

## Abstract

**Purpose:**

Cognitive impairments are frequently reported by cancer survivors. However, these often remain untreated. Understanding the coping strategies used by cancer survivors to manage cognitive impairments offers insights that may inform the development of scalable self-management recommendations and interventions. Consequently, this study therefore explores the specific coping strategies employed by cancer survivors themselves to address cognitive impairments.

**Methods:**

A digital qualitative study with a descriptive design was conducted, including 389 cancer survivors’ responses to an open-ended question in a digital survey: “What have you found helpful in managing cancer-related cognitive impairments?”. Participants were recruited across Norway through social media platforms and e-mails distributed by Norwegian cancer societies and health care organizations. Data were analyzed using qualitative content analysis.

**Results:**

Five themes were identified as important in managing cognitive impairments: (1) challenging the brain, (2) applying tools, (3) receiving support and validation, (4) managing fatigue, and (5) coping with emotional distress.

**Conclusions:**

Cancer survivors employ various strategies to cope with cognitive impairments, including physical exercise, cognitive training, leisure activities, and compensatory tools. Support and validation from significant others play a crucial role, highlighting the importance of increasing knowledge about cognitive impairments after cancer treatment. Holistic interventions that address contributing factors, such as fatigue and emotional well-being, should be prioritized. These insights may inform the development of future self-management recommendations and interventions. Due to methodological limitations related to recruitment, the findings may not be generalizable to the entire target population.

## Introduction

 Cognitive impairments are frequently reported by cancer survivors [1–3] and experienced throughout the entire cancer trajectory [4, 5]. Such impairments are often described as difficulties with remembering information, multitasking, being attentive, and finding words [6]. Moreover, neuropsychological tests show impaired performance in the domains of attention, memory, processing speed, and executive functioning [1, 2, 7]. Cognitive impairments are found to negatively impact cancer survivors’ work performance, social interactions [8–11], quality of life [12], and mental health [3, 13–15]. The negative impact of cancer-related cognitive impairments demonstrates a need to target these.

However, few cancer survivors receive targeted treatment for cognitive impairments [8, 16, 17]. This might be due to limited access to healthcare professionals who are trained in treating cognitive impairments [18]. Supporting cancer survivors in self-managing cognitive impairments might be one solution.

Present qualitative studies show that cancer survivors report using various coping strategies for cognitive impairments, such as physical exercise and games [19–21]. In addition, they also seek emotional support from significant others and participate in cancer support groups [19, 22]. Using tools such as memory aids, reminders, frequent breaks, and writing things down are other coping strategies reported in previous studies [8, 21–23]. However, to develop effective self-management recommendations and interventions, we need more knowledge. Therefore, it is essential to explore the coping strategies used by cancer survivors with cognitive impairments.

Consequently, this study addresses the research question: What coping strategies do cancer survivors use to manage cognitive impairments?

## Methods

### Design

To answer the research question, we conducted a qualitative study with a descriptive design.

### Eligibility criteria and recruitment

The eligibility criteria for this study were as follows: (a) being over the age of 18 and (b) having received cancer treatment. The recruitment of participants took place between October 2023 and January 2024. We used a convenience sample wherein participants were recruited digitally across Norway through various social media platforms, as well as via e-mails distributed by Norwegian cancer patient societies and health care organizations. Participants underwent digital screening to confirm the inclusion criteria.

### Data collection

Qualitative data for this study were obtained from the open-ended question “What have you found helpful in managing cancer-related cognitive impairments?” through a digital survey aiming to investigate aspects of cancer survivors’ experiences with coping strategies for cognitive impairments. Among the 735 individuals who participated in the survey, 389 participants provided a response to the open-ended question and were included in the current study.

### Analysis

Data were analyzed by using content analysis, employing inductive and constructivist approaches aiming to understand the subjective meaning individuals assign to their experiences [24, 25]. NVivo was used for data management. The research team consisted of researchers with clinical expertise in neuropsychology, clinical psychology, and cancer nursing. In addition, representatives from patient organizations participated in the team. Initially, the first and second authors independently read through participants’ responses to familiarize themselves with the dataset and find content relevant to the research question. The first author has a background in neuropsychology, and she recognized that her previous experience with cognitive rehabilitation in other patient groups might influence her interpretation of the data. She therefore remained cautious about the potential impact of this knowledge on the analysis. To ensure reflexivity in the analytical process, the first author’s experiences were discussed with the second author, who had no prior experience in neuropsychology. An initial coding framework was developed to code the data. Meaning units were registered as a single response, allowing for multiple meaning units from each participant. The coding framework was refined iteratively through team discussions. Codes representing similar constructs were integrated, and the condensed meaning codes were reviewed and discussed within the research team to identify themes of shared meaning related to coping strategies for cognitive impairments. The first and second authors, leading the analytical process, revisited the dataset to ensure no significant responses had been overlooked and to assess the richness of the themes.

### Ethics

The study was approved by the Regional Committee for Medical Research Ethics of Western Norway (registration number 619504). Informed consent to participate in the study and for the results of the study to be published was obtained digitally. Data were securely stored in accordance with data protection requirements at Haukeland University Hospital. The study was performed in line with the principles of the Declaration of Helsinki.

## Results

The characteristics of the participants are presented in Table 1.

**Table 1:** Participants’ demographic and clinical characteristics

Variables	*n*
Age	
18–29 years	14
30–39 years	33
40–49 years	105
50–59 years	124
60–65 years	69
> 66 years	44
Gender	
Male	35
Female/other	354
Civil status	
Partner	298
No partner	91
Education	
Higher education	260
No higher education	129
Cancer type	
Breast	202
Gynecological	72
Stomach/bowl	41
Lymphoma	30
Blood and bone marrow	25
Prostate	11
Lung	8
Brain	5
Skin	5
Other	17

Other: mouth and throat, pancreatic, neuroendocrine, anal, bladder, mesothelioma, bone cancer. Some participants had received multiple cancer diagnoses

### Findings

The analysis identified five main themes describing different aspects of coping with cancer-related cognitive impairments: (1) challenging the brain, (2) applying tools, (3) receiving support and validation, (4) managing fatigue, and (5) coping with emotional distress.

#### Theme 1: Challenging the brain

This theme encompassed participants’ experiences with engaging in activities to challenge their cognition as part of their post-treatment trajectory. These activities ranged from using cognitive training apps or solving Rubik’s cubes to the more frequently reported crosswords and sudoku. Participants perceived these cognitively demanding activities as brain exercise, enabling them to stimulate aspects of their cognition, as illustrated by this participant:

I do various tasks such as crosswords, games and sudoku to train my brain and memory.

Furthermore, participants described a variety of approaches to stimulate their cognition, going beyond traditional puzzles such as crosswords and sudoku. Some chose to explore interests that inherently challenge the brain, such as knitting, which demands not only concentration and coordination but also serves as a creative outlet engaging the mind. Similarly, playing musical instruments, reading, learning a new language, or participating in various games all contributed to cognitive enhancement by introducing new challenges and fostering continuous learning. One participant described:

I challenge myself daily to concentrate on different things. Practicing my instrument or listening to an audiobook.

Participants also highlighted the positive impact of physical exercise such as yoga, cardio exercises, weightlifting, balance training, and outdoor activities, on their cognitive functioning. While the primary focus here was not on cognitive training, participants recognized the broader benefits of regular exercise, including its positive impact on brain health, as illustrated in this quote:

My brain only functions normally when I regularly do cardio exercise.

#### Theme 2: Applying tools

This theme illustrated the usefulness of integrating various tools into daily life to cope with cancer-related cognitive impairments. Many participants emphasized the role of smartphones as an indispensable aid in their daily routines. Here, they used functions such as calendars, alarms, and notes to remember important information, as exemplified by one participant:

My mobile phone, computer, and iPad have really helped me out in daily life. Remembering things, keeping track of notes, dates, and future events. These devices are my memory. Freeing energy and minimizing the fear of forgetting completely. It’s sort of like an encyclopaedia.

Participants also expressed a preference for information in written format, as they had experienced challenges with relying solely on oral communication. They emphasized that information provided verbally was often easily forgotten. The act of receiving information in written form was seen as valuable in retaining crucial details, contributing to a sense of control and empowerment in managing their health.

Overall, participants emphasized the broader theme of facilitating their daily lives as a means of enhancing overall well-being. From adopting smartphone applications to reducing noise, participants stressed the importance of employing strategies that streamlined their daily routines. Such facilitation was described as a practical necessity, but also as a significant contributor to reducing stress levels and fostering a positive and controllable experience of everyday life.

#### Theme 3: Receiving support and validation

Participants expressed that it was valuable to receive information about cognitive impairments from healthcare providers. Some participants described positive experiences of receiving support and advice from healthcare providers, as outlined by this participant:

I needed help from professionals to sort out my thoughts and dare to be open about struggling with cognitive problems.

Participants also highlighted the value of having support among significant others to cope with the emotional and practical challenges following their cognitive impairments. The understanding and empathy offered by family, friends, and colleagues were described as essential components in fostering a positive survivorship experience, contributing to emotional resilience and overall well-being:

Receiving support from my husband is vital as he knows me very well. He can help me out when I struggle during conversations and forgetting things.

Participants also expressed the importance of sharing experiences with others who had undergone similar cancer trajectories. They found that the common ground created through shared experiences fostered a unique understanding that transcended conventional support. Participants also found comfort in connecting with individuals who empathized with their emotions, providing a sense of community and shared strength in facing cognitive difficulties, as described by this participant:

After a stay at the rehabilitation centre and talking to others that have breast cancer, I realized that I am not alone with such difficulties.

#### Theme 4: Managing fatigue

This theme underscores the significance of acknowledging fatigue following cancer treatment, as some participants identified fatigue as a contributing factor to cognitive difficulties. Participants shared various strategies to cope with fatigue such as planning activities, reducing demands from themselves or others, minimizing exposure to stimuli, resting, taking breaks, and allowing themselves more time to alleviate stress.

I have identified some physical signs of when I’ve exceeded my limits. I agree with myself that when I feel these signs, I prioritize and schedule periods of rest.

Other approaches to coping with fatigue and facilitating a state of relaxation were mindfulness, meditation, and relaxation techniques. As one participant wrote:

Mindfulness has been the best for me. Thirty minutes daily meditation and I can cope better with daily life challenges.

#### Theme 5: Coping with emotional distress

This theme concerned the emotional toll of grappling with perceived inadequacies, which emerges as a prevalent concern among some participants. They described experiencing negative emotions when faced with cognitively challenging situations. In those moments, they recognized a need for strategies to navigate and manage their emotions effectively, as the emotions might strain their cognition. Participants described that they searched for acceptance, such as acknowledging a changed starting point and adjusting personal demands and expectations accordingly, as one participant noted:

Learn and tolerate negative emotions such as shame, fear, sadness and envy. It’s not dangerous, say hello to the nasty feelings and thoughts, and move on. Don't let them eat you.

## Discussion

This study aimed to identify strategies that cancer survivors use to manage cognitive impairments, which can inform the development of self-management recommendations and interventions.

A key finding of this study was that the participants used various approaches to challenge their cognitive functioning. Cognitive training activities were perceived as helpful strategies for managing cognitive impairments. This aligns with previous qualitative research on coping strategies among cancer survivors [20, 22, 26]. As regards commercial cognitive training apps and doing crosswords, their efficacy in improving cognitive functioning is mixed [27–30]. However, these approaches pose no significant risk or harm and are safe for cancer survivors to use independently. A less conventional approach for challenging cognition that was described by the participants involved engaging in leisure activities such as playing music, knitting, and learning new languages. Research suggests that learning to knit or play the piano may increase cognitive functioning [31, 32]. However, the literature regarding the efficacy of leisure activities on cancer-related cognitive impairments remains scarce. Notwithstanding, tailoring these activities to individual interests might encourage consistent engagement from cancer survivors. Further research is needed to explore the efficacy and feasibility of interventions based on cancer survivors’ personal interest for cognitively stimulating leisure activities. The participants in this study also used physical exercise as a strategy for improving cognitive functioning. While research on the efficacy of physical exercise for cognitive impairments is limited, some studies have shown that exercise can improve cognitive functioning [33, 34]. In Norway, access to physical exercise among cancer survivors is relatively good [35], making it a feasible and resource-efficient approach for addressing cognitive impairments without putting additional strain on current healthcare providers’ resources.

Another finding was that the participants relied on compensatory tools, such as digital applications and note-taking. Similar results have been reported in previous studies [20, 22]. Compensatory tools are relatively easy for cancer survivors to integrate into everyday life, which might explain their widespread use among the participants in this study. However, a study reported that some cancer survivors perceive compensatory tools as a threat to their cognitive functioning, believing that reduced cognitive effort may exacerbate their impairments [23]. Contrary to this belief, interventions that incorporate compensatory approaches have been shown to improve cognitive functioning [36]. By using compensatory techniques, cancer survivors may conserve cognitive resources for other essential tasks in their everyday lives. To enhance the benefits of compensatory tools, healthcare professionals should address concerns about their use. Providing psychoeducation about the positive effect of compensatory approaches can help reassure cancer survivors and encourage their adoption as a tool for self-management.

Participants in our study highlighted the importance of receiving information and validation from healthcare professionals. This concurs with previous research supporting the critical role of receiving social support, understanding, and validation in helping cancer survivors cope with cognitive impairments [20, 22]. Despite this, most cancer survivors report inadequate support from healthcare professionals for cognitive impairments [8, 16, 17]. Healthcare professionals with specialized knowledge of cancer-related cognitive impairments are more likely to offer support [37]. This underscores the importance of enhancing healthcare providers’ understanding of cognitive impairments. Interestingly, even minimal supports, such as unguided, remote, or asynchronous digital therapist support, have been shown to have an effect in other conditions [38, 39]. This suggests that such approaches deserve further exploration in relation to for cancer-related cognitive impairments. Participants also pointed to the crucial role of family, friends, and colleagues in providing support. Educating these significant others might help alleviate some of the burden on healthcare providers.

Some participants experienced a positive effect on cognitive impairments by targeting other symptoms, such as fatigue, by taking breaks and engaging in relaxation techniques. Accepting their cognitive limitations also helped participants reduce negative thought patterns. Fatigue and emotional symptoms are closely associated with reduced cognitive functioning [13, 14]. Given the multifaceted nature of cancer-related cognitive impairments [6], holistic and personalized treatments warrant further investigation [40].

### Strengths and limitations

The main strength of this study is its focus on the coping strategies cancer survivors use for managing cognitive impairments, described in their own words. This approach provides insights for future efforts to address cancer-related cognitive impairments. However, this study has several limitations. Participants were recruited via social media platforms and email, which might have introduced selection bias by excluding proportions of the target group. This recruitment strategy could partly explain the predominance of female participants, as adult women in Norway use social media more frequently than men [40]. Given that a majority of the sample consisted of highly educated women, the findings from this study might not be generalizable to all cancer survivors. Future research should aim to enhance participant diversity by employing alternative recruitment strategies that reach those from a wide range of demographic backgrounds. This is crucial as understanding the perspectives of a diverse target group is essential for developing effective and relevant interventions that are meaningful for the end users. Another limitation concerns the qualitative descriptions from each participant being limited, although this may have been compensated for by the large sample size. More detail might have been obtained by in-depth qualitative interviews.

## Conclusion

This study highlights important strategies for cancer survivors to cope with cognitive impairments such as challenging the brain through physical exercise, cognitive training, and leisure activities. In addition, employing compensatory tools. Moreover, increasing the competence of significant others and healthcare professionals supports cancer survivors in coping. Cancer survivors might also improve cognitive functioning by addressing other symptoms, such as fatigue and distress. These insights may inform the development of future self-management recommendations and interventions for cancer-related cognitive impairments. However, the findings should be interpreted with caution as recruitment through social media platforms may have excluded parts of the target group. To ensure inclusive and effective future interventions, research should also aim to explore how cancer survivors from diverse demographic backgrounds cope with cognitive impairments.

## Data Availability

The data can be requested from the corresponding author subject to the approval of the data protection officer at Haukeland University hospital and the Regional Comité for Health and Research Ethics.

## References

[1] Janelsins MC, Heckler CE, Peppone LJ, Kamen C, Mustian KM, Mohile SG, Magnuson A, Kleckner IR, Guido JJ, Young KL (2017) Cognitive complaints in survivors of breast cancer after chemotherapy compared with age-matched controls: an analysis from a nationwide, multicenter, prospective longitudinal study. J Clin Oncol 35:506–514. 10.1200/JCO.2016.68.582 28029304 10.1200/JCO.2016.68.5826 PMC5455314

[2] Lange M, Joly F, Vardy J, Ahles T, Dubois M, Tron L, Winocur G, De Ruiter M, Castel H (2019) Cancer-related cognitive impairment: an update on state of the art, detection, and management strategies in cancer survivors. Ann Oncol 30:1925–1940. 10.1093/annonc/mdz410 31617564 10.1093/annonc/mdz410 PMC8109411

[3] Schmidt JE, Beckjord E, Bovbjerg DH, Low CA, Posluszny DM, Lowery AE, Dew MA, Nutt S, Arvey SR, Rechis R (2016) Prevalence of perceived cognitive dysfunction in survivors of a wide range of cancers: results from the 2010 LIVESTRONG survey. J Cancer Surviv 10:302–311. 10.1007/s11764-015-0476-5 26238504 10.1007/s11764-015-0476-5 PMC5772767

[4] Janelsins MC, Kesler SR, Ahles TA, Morrow GR (2014) Prevalence, mechanisms, and management of cancer-related cognitive impairment. Int Rev Psychiatry 26:102–113. 10.3109/09540261.2013.864260 24716504 10.3109/09540261.2013.864260 PMC4084673

[5] Vardy JL, Dhillon HM, Pond GR, Rourke SB, Bekele T, Renton C, Dodd A, Zhang H, Beale P, Clarke S (2015) Cognitive function in patients with colorectal cancer who do and do not receive chemotherapy: a prospective, longitudinal, controlled study. J Clin Oncol 33:4085–4092. 10.1200/JCO.2015.63.09 26527785 10.1200/JCO.2015.63.0905 PMC5683012

[6] Ahles TA, Root JC (2018) Cognitive effects of cancer and cancer treatments. Annu Rev Clin Psychol 14:425–451. 10.1146/annurev-clinpsy-050817-084903 29345974 10.1146/annurev-clinpsy-050817-084903 PMC9118140

[7] Lindner OC, Phillips B, McCabe MG, Mayes A, Wearden A, Varese F, Talmi D (2014) A meta-analysis of cognitive impairment following adult cancer chemotherapy. Neuropsychology 28:726–740. 10.1037/neu0000064 24635712 10.1037/neu0000064 PMC4143183

[8] Boykoff N, Moieni M, Subramanian SK (2009) Confronting chemobrain: an in-depth look at survivors’ reports of impact on work, social networks, and health care response. J Cancer Surviv 3:223–232. 10.1007/s11764-009-0098-x 19760150 10.1007/s11764-009-0098-x PMC2775113

[9] Henderson FM, Cross AJ, Baraniak AR (2019) ‘A new normal with chemobrain’: experiences of the impact of chemotherapy-related cognitive deficits in long-term breast cancer survivors. Health Psychol 6:1–10. 10.1177/2055102919832234 10.1177/2055102919832234 PMC6405778 30873289

[10] Lange M, Licaj I, Clarisse B, Humbert X, Grellard JM, Tron L, Joly F (2019) Cognitive complaints in cancer survivors and expectations for support: results from a web–based survey. Cancer Med 8:2654–2663. 10.1002/cam4.2069 30884207 10.1002/cam4.2069 PMC6536919

[11] Von Ah D, Storey S, Crouch A (2018) Relationship between self-reported cognitive function and work-related outcomes in breast cancer survivors. J Cancer Surviv 12:246–255. 10.1007/s11764-017-0664-6 29222703 10.1007/s11764-017-0664-6

[12] Maeir T, Nahum M, Makranz C, Tsabari S, Peretz T, Gilboa Y (2023) Predictors of quality of life among adults with self-reported cancer related cognitive impairment. Disabil Rehabil 45:1056–1062. 10.1080/09638288.2022.2050954 35297702 10.1080/09638288.2022.2050954

[13] Castellon SA, Ganz PA, Bower JE, Petersen L, Abraham L, Greendale GA (2004) Neurocognitive performance in breast cancer survivors exposed to adjuvant chemotherapy and tamoxifen. J Clin Exp Neuropsychol 26:955–969. 10.1080/13803390490510905 15742545 10.1080/13803390490510905

[14] Vearncombe KJ, Rolfe M, Wright M, Pachana NA, Andrew B, Beadle G (2009) Predictors of cognitive decline after chemotherapy in breast cancer patients. J Int Neuropsychol Soc 15:951–962. 10.1017/S1355617709990567 19709455 10.1017/S1355617709990567

[15] Yang Y, Hendrix CC (2018) Cancer-related cognitive impairment in breast cancer patients: influences of psychological variables. Asia Pac J Oncol Nurs 5:296–306. 10.4103/apjon.apjon_16_18 29963592 10.4103/apjon.apjon_16_18 PMC5996591

[16] Bolton G, Isaacs A (2018) Women’s experiences of cancer-related cognitive impairment, its impact on daily life and care received for it following treatment for breast cancer. Psychol Health Med 23:1261–1274. 10.1080/13548506.2018.1500023 30048158 10.1080/13548506.2018.1500023

[17] Von Ah D, Habermann B, Carpenter JS, Schneider BL (2013) Impact of perceived cognitive impairment in breast cancer survivors. Eur J Oncol Nurs 17:236–241. 10.1016/j.ejon.2012.06.002 22901546 10.1016/j.ejon.2012.06.002

[18] Janelsins MC, Van Dyk K, Hartman SJ, Koll TT, Cramer CK, Lesser GJ, Barton DL, Mustian KM, Wagner LI, Ganz PA (2024) The National Cancer Institute clinical trials planning meeting to address gaps in observational and intervention trials for cancer-related cognitive impairment. JNCI J Nat Cancer Instit djae209. 10.1093/jnci/djae209 10.1093/jnci/djae209 PMC11807440 39250738

[19] Cheung Y, Shwe M, Tan Y, Fan G, Ng R, Chan A (2012) Cognitive changes in multiethnic Asian breast cancer patients: a focus group study. Ann Oncol 23:2547–2552. 10.1093/annonc/mds029 22396443 10.1093/annonc/mds029

[20] Myers JS (2010) Chemotherapy-related cognitive impairment: the breast cancer experience. Dissertation, University of Kansas. 10.1188/12.ONF.E31-E40 22201666

[21] Tometich DB, Welniak T, Gudenkauf L, Maconi ML, Fulton HJ, Martinez Tyson D, Zambrano K, Hasan S, Rodriguez Y, Bryant C (2024) I couldn’t connect the wires in my brain. Young adult cancer survivors' experience with cognitive functioning. Psycho‐Oncology 33:e6309. 10.1002/pon.6309 10.1002/pon.6309 PMC11249037 38420860

[22] Munir F, Kalawsky K, Lawrence C, Yarker J, Haslam C, Ahmed S (2011) Cognitive intervention for breast cancer patients undergoing adjuvant chemotherapy: a needs analysis. Cancer Nurs 34:385–392. 10.1097/NCC.0b013e31820254f3 21242770 10.1097/NCC.0b013e31820254f3

[23] Chapman B, Derakshan N, Grunfeld EA (2022) Exploring primary breast cancer survivors’ self-management of sustained cancer-related cognitive impairment in the workplace. Psychooncology 31:606–613. 10.1002/pon.5844 34699652 10.1002/pon.5844

[24] Thorne S, Kirkham SR, MacDonald-Emes J (1997) Interpretive description: a noncategorical qualitative alternative for developing nursing knowledge. Res Nurs Health 20:169–177. 10.1002/(SICI)1098-240X(199704)20:2%3c169::AID-NUR9%3e3.0.CO;2-I 9100747 10.1002/(sici)1098-240x(199704)20:2<169::aid-nur9>3.0.co;2-i

[25] White MD, Marsh EE, Marsh EE, White MD (2006) Content analysis: a flexible methodology. Libr Trends 55:22–45. 10.1353/lib.2006.0053

[26] Sharma S, Brunet J (2023) Young adults’ lived experiences with cancer-related cognitive impairment: an exploratory qualitative study. Curr Oncol 30:5593–5614. 10.3390/curroncol30060422 37366905 10.3390/curroncol30060422 PMC10297401

[27] Bray VJ, Dhillon HM, Bell ML, Kabourakis M, Fiero MH, Yip D, Boyle F, Price MA, Vardy JL (2017) Evaluation of a web-based cognitive rehabilitation program in cancer survivors reporting cognitive symptoms after chemotherapy. J Clin Oncol 35:217–225. 10.1200/JCO.2016.67.82 28056205 10.1200/JCO.2016.67.8201

[28] Damholdt MF, Mehlsen M, O’Toole MS, Andreasen RK, Pedersen AD, Zachariae R (2016) Web-based cognitive training for breast cancer survivors with cognitive complaints—a randomized controlled trial. Psychooncology 25:1293–1300. 10.1002/pon.4058 26763774 10.1002/pon.4058 PMC5111748

[29] Kesler S, Hosseini SH, Heckler C, Janelsins M, Palesh O, Mustian K, Morrow G (2013) Cognitive training for improving executive function in chemotherapy-treated breast cancer survivors. Clin Breast Cancer 13:299–306. 10.1016/j.clbc.2013.02.004 23647804 10.1016/j.clbc.2013.02.004 PMC3726272

[30] Janette LV, Victoria JB, Haryana MD (2017) Cancer-induced cognitive impairment: practical solutions to reduce and manage the challenge. Future Oncol 13:767–771. 10.2217/fon-2017-0027 28266247 10.2217/fon-2017-0027

[31] Hartzell JW, Yaguda S, Boselli D (2021) Knitting to improve cognition and reduce stress in cancer survivors: a pilot study. J Clin Oncol 30. 10.1200/JCO.2021.39.15_suppl.e24049

[32] Rodriguez-Wolfe M, Anglade D, Gattamorta KA, Hurwitz WB, Pirl WF (2019) Individualized piano instruction for improving cognition in breast cancer survivors. Oncol Nurs Forum 46:605–615. 10.1188/19.ONF.605-615 31424459 10.1188/19.ONF.605-615

[33] Bender CM, Sereika SM, Gentry AL, Cuglewski C, Duquette J, Grove G, Cummings M, Mg C, Brufsky AM, McAuliffe P (2024) Effects of aerobic exercise on neurocognitive function in postmenopausal women receiving endocrine therapy for breast cancer: the Exercise Program in Cancer and Cognition randomized controlled trial. Psychooncology 33:e6298. 10.1002/pon.6298 38911475 10.1002/pon.6298 PMC11189639

[34] Campbell KL, Zadravec K, Bland KA, Chesley E, Wolf F, Janelsins MC (2020) The effect of exercise on cancer-related cognitive impairment and applications for physical therapy: systematic review of randomized controlled trials. Phys Ther 100:523–542. 10.1093/ptj/pzz090 32065236 10.1093/ptj/pzz090 PMC8559683

[35] Nilsen JV, Jensen L, Bohn NM, Øverli B (2018) Å leve med og etter kreft. Norwegian Cancer Society. https://kreftforeningen.no/content/uploads/2023/11/rehabiliteringsrapporten-2018-web.pdf . Accessed 11 March 2025

[36] Vance DE, Frank JS, Bail J, Triebel KL, Niccolai LM, Gerstenecker A, Meneses K (2017) Interventions for cognitive deficits in breast cancer survivors treated with chemotherapy. Cancer Nurs 40:E11–E27. 10.1097/NCC.0000000000000349 26918390 10.1097/NCC.0000000000000349

[37] He S, Lim CYS, Dhillon HM, Shaw J (2022) Australian oncology health professionals’ knowledge, perceptions, and clinical practice related to cancer-related cognitive impairment and utility of a factsheet. Support Care Cancer 30:4729–4738. 10.1007/s00520-022-06868-z 35122530 10.1007/s00520-022-06868-z PMC9046357

[38] Akdemir A, Smith AB, Wu VS, Rincones O, Russell H, Lyhne JD, Kemp E, David M, Bamgboje-Ayodele A (2024) Guided versus non-guided digital psychological interventions for cancer patients: a systematic review and meta-analysis of engagement and efficacy. Psychooncology 33:e6290. 10.1002/pon.6290 38282223 10.1002/pon.6290

[39] Tong L, Panagiotopoulou O-M, Cuijpers P, Karyotaki E (2024) The effectiveness of self-guided interventions in adults with depressive symptoms: a systematic review and meta-analysis. EBioMedicine 105:1–10. 10.1016/j.ebiom.2024.105208 10.1016/j.ebiom.2024.105208 PMC11226978 38876043

[40] Binarelli G, Duivon M, Joly F, Ahmed-Lecheheb D, Lange M (2023) Cancer-related cognitive impairment: current perspectives on the management of cognitive changes following cancer treatment. Expert Rev Neurother 23:249–268. 10.1080/14737175.2023.2187288 36951414 10.1080/14737175.2023.2187288

[41] Bekkengen VB (2024) The Norwegian Media Barometer. Office for National Statistics. https://www.ssb.no/kultur-og-fritid/tids-og-mediebruk/artikler/norsk-mediebarometer-2024. Accessed 23 June 2025.

